# The Cure and Culture of Children

**Published:** 1880-12

**Authors:** 


### BIBLIOGRAPHICAL.

The Cure ane Culture of Children.—A practical treatise for the use of parents. By Thos. S. Sozinskey, M.D., Ph. D., Author of "The Culture of Beauty, etc., etc. H. C. Watts & Co., Phila. 1880.

This is, as it purports to be, a practical treatise on the care and culture of children, and is directed more particularly to parents. Of no small moment, however, is this question to the medical profession.

The author deals extensively in the principles of hygiene, and very properly so, too, since upon the proper application of these much of the child's moral, mental and physical perfection depends.

Mothers who would hope to acquire a fund of precept and experience in rearing children on practical and scientific principles, will find this a very valuable work.

